# Sexually dimorphic role of the locus coeruleus PAC1 receptors in regulating acute stress-associated energy metabolism

**DOI:** 10.3389/fnbeh.2022.995573

**Published:** 2022-10-05

**Authors:** Samuel J. Duesman, Sanutha Shetty, Sanil Patel, Neha Ogale, Farzanna Mohamed, Njeri Sparman, Prashant Rajbhandari, Abha Karki Rajbhandari

**Affiliations:** ^1^Departments of Psychiatry and Neuroscience, Icahn School of Medicine at Mount Sinai, New York, NY, United States; ^2^Diabetes, Obesity, and Metabolism Institute, Icahn School of Medicine at Mount Sinai, New York, NY, United States

**Keywords:** locus coeruleus, energy expenditure, brown adipose fat tissue, stress, PACAP, PAC1, metabolism

## Abstract

Severe stress leads to alterations in energy metabolism with sexually dimorphic onset or severity. The locus coeruleus (LC) in the brainstem that mediates fight-or-flight-or-freeze response to stress is sexually dimorphic in morphology, plays a key role in interactions between diet and severe stressors, and has neuronal input to the brown adipose tissue (BAT)—a thermogenic organ important for energy balance. Yet, little is known on how LC coordinates stress-related metabolic adaptations. LC expresses receptors for the neuropeptide PACAP (pituitary adenylate cyclase activating peptide) and PACAP signaling through PAC1 (PACAP receptor) are critical regulators of various types of stressors and energy metabolism. We hypothesized that LC-PAC1 axis is a sex-specific central “gatekeeper” of severe acute stress-driven behavior and energy metabolism. Selective ablation of PAC1 receptors from the LC did not alter stress response in mice of either sex, but enhanced food intake in females and was associated with increased energy expenditure and BAT thermogenesis in male mice. These results show a sexually dimorphic role of the LC-PAC1 in regulating acute stress-related energy metabolism. Thus, by disrupting LC-PAC1 signaling, our studies show a unique and previously unexplored role of LC in adaptive energy metabolism in a sex-dependent manner.

## Introduction

Severe stressors lead to behavior and metabolic dysfunctions with a sexually dimorphic etiology (Dallman et al., 2003; Dedert et al., 2010; Nowotny et al., 2010; Farr et al., 2014; Levine et al., 2014; LeardMann et al., 2015; Michopoulos et al., 2016; Wolf et al., 2016). Understanding neural mechanisms of stress-related energy metabolism can allow development of novel ways of balancing optimal energy metabolism to reduce maladaptive impact on health and well-being (Breslau, 2002; Raikkonen et al., 2002; Dobie et al., 2004; Tolin and Foa, 2006; Heppner et al., 2009; Bell et al., 2011; Roenholt et al., 2012; Kubzansky et al., 2014; Udo et al., 2014; Buta et al., 2018; Pooley et al., 2018; American Psychological Association, 2020; Escarfulleri et al., 2021; Kautzky et al., 2021).

Locus coeruleus (LC) coordinates stress-associated adaptive/maladaptive arousal (alertness via enhanced blood pressure, heart rate, and breathing), “fight-or flight-or freeze” defensive responses, and energy metabolism (Aston-Jones et al., 1999; Aston-Jones and Cohen, 2005; Jovanovic et al., 2010; Fullana et al., 2016; Mothersill and Donohoe, 2016; Borodovitsyna et al., 2018; Kral et al., 2018; Kim et al., 2019). LC is a major source of norepinephrine (NE) to the entire forebrain axis and is a sexually dimorphic structure in morphology, gene expression, and stress responsiveness (Curtis et al., 2006; Samuels and Szabadi, 2008; Bangasser et al., 2016; Mulvey et al., 2018). LC activity and energy metabolism are causally linked, whereby enhanced LC activity decreases feeding, increases body temperature and oxygen consumption, while LC lesion decreases weight gain (Guimaraes et al., 2013). Furthermore, activation of ATP-dependent potassium channel in LC increases epididymal fat, while binge eating, or consumption of palatable foods, decrease LC activity (Guimaraes et al., 2013; Bello et al., 2014). LC also has functional connectivity to the brown adipose tissue (BAT)—a critical determinant of systemic energy balance via sympathetic activation and induction of thermogenic factors such as mitochondrial uncoupling protein 1 (UCP1) (Aston-Jones et al., 1994; Canale et al., 2013; Thorp and Schlaich, 2015; Atzori et al., 2016; Rabasa and Dickson, 2016; Caron et al., 2018; Naegeli et al., 2018; Rabasa et al., 2019; Morris et al., 2020; Yang et al., 2021), which is primarily expressed in brown adipocytes and uncouples oxidative phosphorylation from ATP synthesis to generate heat (Rousset et al., 2004; Fedorenko et al., 2012). LC inhibition reduces BAT thermogenesis and markedly attenuates BAT sympathetic activity independent of cold stress activity (Almeida et al., 2004). Despite clinical or preclinical literature strongly informing that trauma-like stressors contribute to the onset, maintenance, or aggravation of metabolic dysfunctions potentially via LC sympathetic pathways (Pervanidou and Chrousos, 2012), knowledge of combined behavioral and metabolic functions via LC neuromodulation is incomplete due to poor consideration of sex differences in animal models of severe stressors. Given that LC is sexually dimorphic in morphology and regulates stress-associated behavioral changes, we predicted that LC mediates long-lasting metabolic consequences to severe stressors, thereby acting as a switch for arousal and energy mobilization in humans and animals in a sexually dimorphic manner (Cannon, 1932; Southwick et al., 1999; O’Donnell et al., 2004; Borodovitsyna et al., 2018; Li et al., 2018; Grueschow et al., 2021). Therefore, by using a robust model of trauma-like stressor, we tested effects on whole body energy metabolism and BAT thermogenesis via a novel approach of LC neuropeptidergic modulation.

Several neuropeptides like the corticotrophin releasing factor are known to regulate LC functions. However, the receptor PAC1 (gene name *ADCYAPR1*), which is selective for the neuropeptide PACAP (gene name *ADCYAP1*), is highly expressed in the LC. Yet, PACAP and PAC1’s role in stress and metabolism has not been previously explored. LC-PAC1 has been shown to be important for regulating somatic symptoms associated with morphine withdrawal (Otto et al., 2001; Martin et al., 2010; Zhang et al., 2021). Human genetic studies have linked PACAP/PAC1 to post-traumatic stress disorder (PTSD) diagnosis and symptom severity. Specifically, mutations to the PAC1 gene are associated with PTSD symptom severity in women (Ressler et al., 2011). PACAP/PAC1 signaling are also sex-specific sympathetic regulators of a variety of stressors, energy homeostasis, mood, feeding, appetite, and metabolism (Beck, 2000; D’Este et al., 2000; Otto et al., 2001; Lein et al., 2007; Vaudry et al., 2009; Boughton and Murphy, 2013; Bangasser et al., 2016; Gastelum et al., 2021; Zhang et al., 2021). PACAP knockout early in development is embryonically lethal due to respiratory or metabolic alterations suggesting its biological importance for survival (Miyata et al., 1990; Hosoya et al., 1992; Gray et al., 2001; Cummings et al., 2004). PAC1 knockout in adult mice causes deficits in lipid metabolism, increase in serum triglycerides, fatty acids, cholesterol, and leptin indicating their role in energy metabolism (Gray et al., 2001; Adams et al., 2008; Hawke et al., 2009; Rudecki and Gray, 2016; Bozadjieva-Kramer et al., 2021; Chang et al., 2021; Filatov et al., 2021; Maunze et al., 2022).

We previously reported that amygdalar PACAP/PAC1 signaling regulates fear behaviors in a sexually dimorphic manner (Rajbhandari et al., 2021). Here, we aimed to unravel the role of LC-PAC1 on severe stress-associated energy homeostasis at the level of: (i) behavioral stress response, (ii) whole-body energy expenditure, and (iii) BAT thermogenesis.

## Results

### LC-PAC1 does not mediate sex-dependent regulation of stressor memory

**FIGURE 1:**
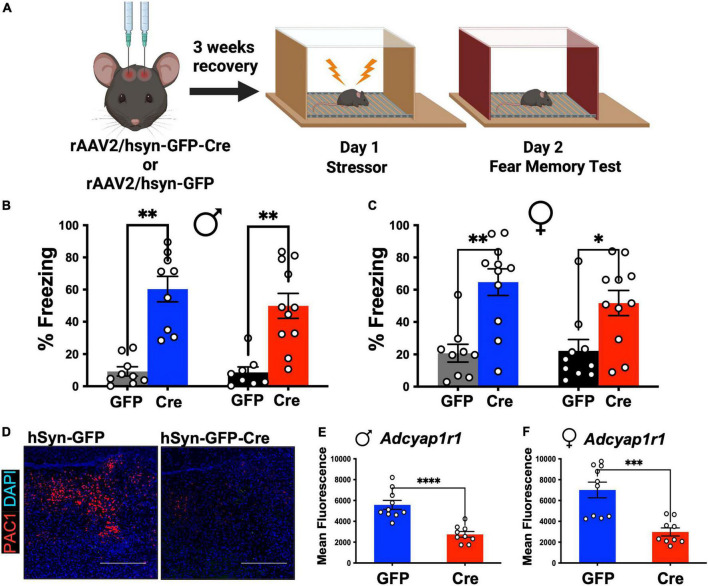
LC-PAC1 expression does not affect memory of the stressor test. **(A)** Animals were bilaterally injected with either hSyn-GFP or hSyn-GFP-Cre into the LC. After a 3 week recovery period, animals were run through a contextual fear protocol. **(B,C)** Prior stressors significantly increased freezing in all groups, but no significant effect of LC-PAC1 deletion were observed in memory of the stressor in either males **(B)** or females **(C)**. *N* = 8–11/group. **(D)** Representative RNAScope *in situ* hybridization image of merged PAC1 mRNA and DAPI levels in rAAV2-hSyn-GFP and rAAV2-hSyn-GFP-Cre mice. **(E,F)** Mean fluorescence of PAC1 mRNA intensity in LC of indicated mouse groups. Injection of rAAV2-hSyn-GFP-Cre into the LC resulted in a significant reduction of mean fluorescence of PAC1 mRNA in both males and females. *N* = 9–10/group. *p < 0.05; ^**^p < 0.01. Scale bar = 1 mm.

To comprehensibly determine the role of LC-PAC1 in fear, we followed a detailed experimental timeline and setup (Figure 1A). At 12 weeks of age, we conducted stereotaxic surgeries to microinfuse AAV2-hsyn-GFP-Cre or AAV2-hsyn-GFP into LC (coordinates L/M: ±1; A/P: −5.4; D/V: −4.2) for efficient Cre mediated PAC1 knockdown in PAC1^loxp/loxp^ mice. Following a 3-week recovery period to allow for viral transfection, mice received 10 foot shocks on Day 1 as the stressor experience. This protocol produces a long-lasting fear in rodents (Rau and Fanselow, 2009; Rajbhandari et al., 2018). Two way ANOVA did not reveal effect of sex [*F*(1, 70) = 2.493, *p* > 0.05] or interaction [*F*(3,70) = 0.32, *p* > 0.81). There was a main effect of treatment condition [*F*(3, 70) = 24.11, *p* < 0.05] (Figure 1B). Post-hoc analysis revealed that in both males and females freezing was increased (*p* < 0.05) on day 2 compared to unstressed animals regardless of the level of PAC1 receptors in the LC (Figures 1B,C). However, there was no significant effect of LC-PAC1 deletion on freezing for both males and females (*p* > 0.05) (Figures 1B,C). The deletion of PAC1 receptors was verified using RNAScope mRNA *in situ* hybridization (see section “Materials and methods”) for analyzing expression of PAC1 mRNA, *Adcyap1r1*, in tissue sections. We conducted fluorescence microscopy on mRNA puncta of PAC1 and represented as mean fluorescence (Figure 1D). As shown in Figures 1D–F, mean fluorescence of *Adcyap1r1* was measured for each image and showed a significant decrease in Cre injected male and female mice compared to GFP-only control.

### LC ablation of PAC1 mediates sex-dependent regulation of stress-associated whole-body energy metabolism

**FIGURE 2:**
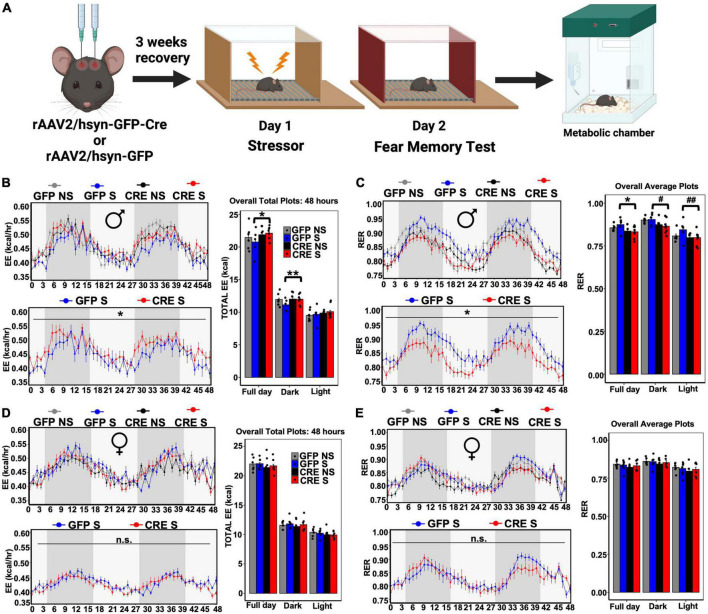
LC-PAC1 deletion results in sexually dimorphic stress-induced metabolic changes. **(A)** Animals were bilaterally injected with either hSyn-GFP or hSyn-GFP-Cre into the LC. After a 3 week recovery period, animals were run through a contextual fear protocol followed by 72 h in the metabolic chamber. **(B,C)** In male mice with LC-PAC1 deletion energy expenditure (EE, kcal/h) was significantly increased **(B)** and respiratory exchange ratio (RER) was significantly reduced **(C)** as evidenced by analysis in Sable Promethion metabolic chambers (12 h light/dark cycle, 48 h total duration, white bar represent light cycle and grey bar represent night cycle). **(D,E)** No significant differences in energy expenditure (EE, kcal/h) **(D)** and respiratory exchange ratio (RER) **(E)** in females. Analysis was performed in Sable Promethion metabolic chambers (12 h light/dark cycle, 48 h total duration, white bar represent light cycle and gray bar represent night cycle. For each of these variables a line graph and bar graph comparing all four groups (hSyn-Cre S, hSyn-Cre NS, hSyn-GFP S, and hSyn-GFP NS) as well as a line graph comparing stressed groups (hSyn-Cre S and hSyn-GFP S) are displayed. *N* = 9–10/group. *p < 0.05; **p < 0.01, ^#^*p* = 0.05, ^##^*p* = 0.07. GFP NS, hSyn-GFP No Shock; GFP S, hSyn-GFP Shock; CRE NS, hSyn-Cre No Shock; and CRE S, hSyn-Cre Shock.

To determine the role of LC-PAC1 deletion in stress-induced energy metabolism, we performed indirect calorimetry using metabolic chambers in stressed or unstressed mice for 72 h (Figure 2A). Mice were acclimatized to individual metabolic chambers for the first 24 h, during which all the groups showed heightened calorimetric data possibly due to stress in a novel context (Supplementary Figures 1A–L). Therefore, we removed the first 24 h data from our calculation. Results of an ANCOVA and ANOVA test on the rest 48 h showed a main effect of LC-PAC1 deletion in stressed males (Supplementary Table 1). Post-hoc analysis showed that stressed males with LC-PAC1 knockdown (CRE S) had a total respiratory exchange ratio (RER) that was significantly lower, total energy expenditure (EE), oxygen consumption (VO2), and carbon dioxide production (VCO2) was significantly higher than stressed males with intact PAC1 receptors (GFP S) in the LC (hSyn-Cre S versus hSyn-GFP S; *p* < 0.05) (Figures 2B,C and Supplementary Figures 2A,B). The results of an ANCOVA and ANOVA showed no significant effect of PAC1 receptor depletion in the LC on food consumption or locomotion were observed between stressed males, respectively (hSyn-Cre S versus hSyn-GFP S; *p* > 0.05) (Supplementary Figures 2C,D and Supplementary Table 1). Interestingly, our results show that GFP S mice showed an overall decrease in EE and increase in RER than other groups (Figures 2B,C), implicating a possible role of LC-PAC1 in attenuating stress-induced increase in energy metabolism. For females, results of an ANCOVA showed that female mice with PAC1 deletion and acute stressor (CRE S) showed no differences in RER, EE, VO2, and VCO2 (Figures 2D,E, Supplementary Figures 2E,G, and Supplementary Table 2) compared to stressed females with intact PAC1 receptors (GFP S). Females showed enhanced total daily food intake compared to mice without deletion or the ones that only received the stress experience (hSyn-Cre S versus hSyn-GFP S; *p* < 0.05) (Supplementary Figure 2H and Supplementary Table 2). No significant differences in locomotor activity were observed within each group in male or female mice (hSyn-Cre S versus hSyn-GFP S; *p >* 0.05), or across sexes (Supplementary Figures 2C,G and Supplementary Tables 1, 2).

### LC-PAC1 regulates stress-associated BAT *Ucp1* expression

**FIGURE 3:**
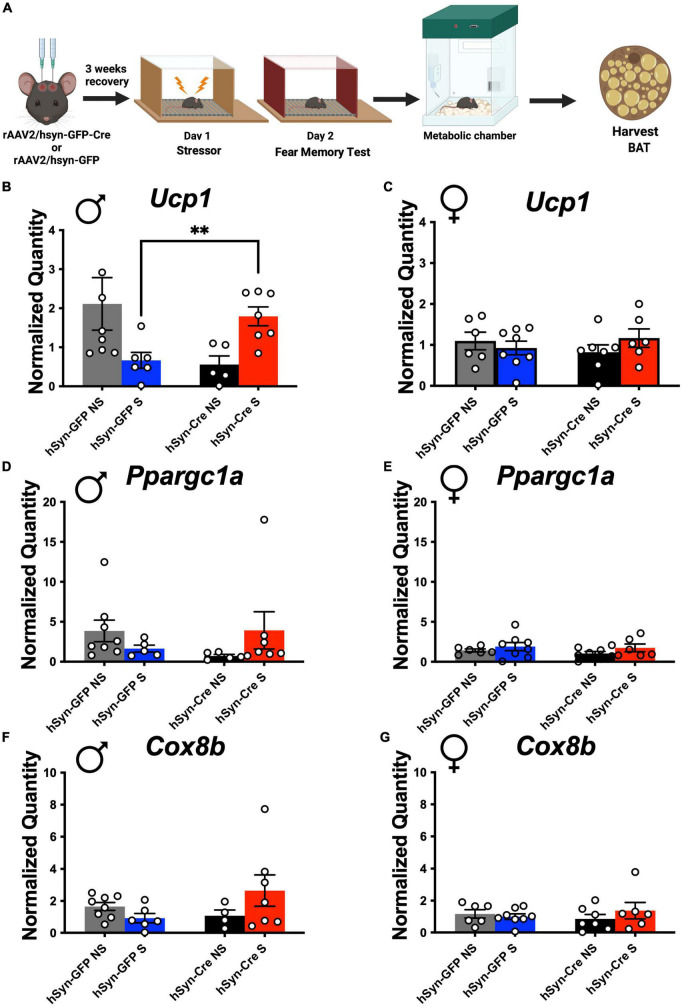
PAC1 deletion in the LC result in a stress-induced thermogenic genetic expression increase in males. **(A)** Animals were bilaterally injected with either hSyn-GFP or hSyn-GFP-Cre into the LC. After a 3-week recovery period, animals were run through a contextual fear protocol followed by 72 h in the metabolic chamber and BAT harvest. **(B–G)** Real-time qPCR of indicated genes from male and female BAT of indicated mouse groups. Stressed male mice with LC-PAC1 deletion showed a significant upregulation in *Ucp1* compared to stressed males with intact LC-PAC1 **(B)**. No significant differences in *Ucp1* expression were observed in females **(C)**. There were no significant differences in expression of *PPARGC1a* in males **(D)** or females **(E)**. No significant differences in expression of *Cox8b* were observed in males **(F)** or females **(G)**. N = 9–10/group. **p < 0.01. *Ucp1*, Uncoupling protein 1; *PPARa*, peroxisome proliferator-activated receptor; *Cox8b*, cytochrome c oxidase, subunit VIIIb; *Elovl3*, elongation of very long chain fatty acids-like 3; *Clstn3*, Calsyntenin 3; *PPARGC1a*, Peroxisome proliferator-activated receptor-gamma coactivator-1 alpha.

Mitochondrial uncoupling by UCP1 is a critical determinant of EE (Aston-Jones et al., 1994; Canale et al., 2013; Thorp and Schlaich, 2015; Atzori et al., 2016; Rabasa and Dickson, 2016; Caron et al., 2018; Naegeli et al., 2018; Rabasa et al., 2019; Morris et al., 2020; Yang et al., 2021). Following indirect calorimetry, stressed and non-stressed mice with and without LC-PAC1 deletion were sacrificed under 3% isoflurane anesthesia, and intrascapular BAT was collected (Figure 3A). RNA purification, cDNA synthesis, and RT-qPCR were performed on BAT samples and expression of genes involved in mitochondrial function, biogenesis, and thermogenesis were measured. Two Way ANOVA revealed no effect of sex and condition [*F*(1,22) = 1.94, *p* > 0.05) on the thermogenic gene *Ucp1 in non-stressed mice*. There was a main effect of sex and condition [*F*(1,9) = 6.462, *p* = 0.0316] on *Ucp1 in stressed mice*. Post-hoc analysis showed that there was a significant increase in thermogenic gene, *Ucp1* expression in stressed LC-PAC1 knockout males compared to stressed males with intact LC-PAC1 (hSyn-Cre S versus hSyn-GFP S; *p* < 0.05) (Figure 3B). Genes encoding proteins important for mitochondrial function such as *Ppargc1a* and *Cox8B* were also increased (not significant) in male CRE S mice compared to GFP S mice (Figures 3D–G). Other BAT genes such as *PPARa*, *Elovl3*, *Dio2*, *Clstn3* were comparable between CRE S and GFP S male mice (Supplementary Figures 3A–H). No significant differences were observed in BAT expression of *Ucp1*, *PPARa*, *Cox8b*, *Elovl3*, *Dio2*, *Clstn3*, or *PPARGC1a* in females (Figure 3C and Supplementary Figures 3A–H). Our BAT analysis supports our indirect calorimetric data, suggesting that ablation of PAC1 receptors in LC of male mice increases BAT thermogenic function in stressed mice which could potentially led to increase in EE.

### Validation of PAC1 receptor deletion from locus coeruleus

**FIGURE 4:**
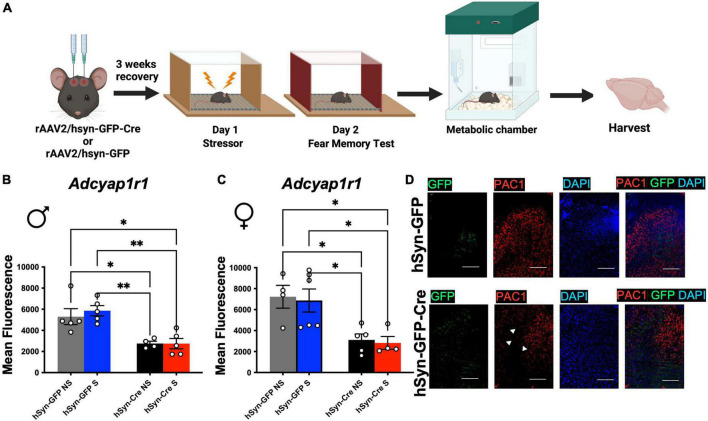
Injection of rAAV2-hSyn-Cre results in a sustained reduction of PAC1 receptors in PAC1^loxP/loxP^ mice. **(A)** Animals were bilaterally injected with either hSyn-GFP or hSyn-GFP-Cre into the LC. After a 3 week recovery period, animals were run through a contextual fear protocol followed by 72 h in the metabolic chamber and brain harvest. **(B,C)** Mean fluorescence of PAC1 mRNA intensity in LC of indicated mouse groups. Injection of rAAV2-hSyn-GFP-Cre into the LC resulted in a significant reduction of mean fluorescence of PAC1 mRNA in both males **(B)** and **(C)** females. **(D)** Representative RNAScope *in situ* hybridization image of PAC1 mRNA, GFP mRNA, DAPI, and merged levels in rAAV2-hSyn-GFP and rAAV2-hSyn-GFP-Cre mice. *p < 0.05; ^**^p < 0.01. Scale bar = 1 mm.

To test if the behavior and energy metabolism changes were due to sustained PAC1 deletion in LC, after the behavior and metabolic tests were completed (Figure 4A), mice were sacrificed, and their brains extracted and immediately stored in at −80^°^C. The brains were sliced at 20 microns in a cryostat and slices containing the LC were collected on microscope slides. The deletion of PAC1 receptors was verified using RNAScope for analyzing expression of RNA in tissue sections. We conducted fluorescence microscopy on mRNA puncta of PAC1 and GFP mRNA and represented as mean fluorescence (Figures 4B–D). Mean fluorescence of *Adcyap1r1* was measured for each image and showed a significant decrease in Cre injected male and female mice compared to GFP-only control (Figures 4B–D). Our data show that sexually dimorphic metabolic perturbation seen in mice are due to acute stress paired with sustained PAC1 deletion in the LC regions.

## Discussion

Our results for the first time show that deletion of PAC1 receptors from the LC does not alter fear expression in male or female mice. However, PAC1 knockdown in the LC of male mice led to an increase in EE and decreased RER. PAC1 ablation from LC in female mice significantly enhanced food intake. We also show that PAC1 deletion in the LC in male mice increased the Ucp1 gene, a measure of BAT thermogenic gene program. Overall, these results show a causal role of LC PAC1 receptors in regulating energy metabolism in a sex-specific manner.

Measurement of stressor memory in mice with and without PAC1 deletion in the LC indicates that these receptors in the LC are not involved in fear regulation. Both male and female groups showed similar levels of freezing with and without PAC1 deletion after acute stressor. It is possible that these receptors are involved in other behavioral measures of stress-exposure including fear extinction, grooming, rearing, and other measures including endocrine measurements such as corticosterone and catecholamines, which will require further studies.

Our results on the whole-body energy metabolism showed a sexually dimorphic effect of LC-PAC1 knockout. Male GFP-only control showed reduced VO2, VCO2, EE and increased RER (indicative of decreased fat oxidation and utilization) compared with male mice with PAC1 deletion and acute stressor. These results were not attributable to changes in locomotion. Female mice with PAC1 deletion in the LC and acute stressor showed enhanced food consumption compared to GFP-only control mice. Analysis of BAT tissue also showed that male mice with PAC1 deletion in the LC and acute stress exposure showed enhanced levels of the *Ucp1* gene compared to mice without PAC1 deletion that went through acute stressor. This indicates that -LC-PAC1 receptors in male mice regulates energy and fat metabolism but in female mice food intake, which is in line with a recent study has showed that LC inhibition increases food intake in response to acute stressor (Sciolino et al., 2019). These findings indicate that under stressor, PAC1 receptors in the LC are important regulators of metabolic functions, whereby removal of these receptors in female mice increase food intake but in male mice alter the peripheral regulation of energy metabolism.

Based on published reports that show LC neurons innervate the BAT (Morrison and Madden, 2014; Wiedmann et al., 2017) and given that LC is sexually dimorphic in morphology, we predict that LC-PAC1 expressing neuronal innervations to BAT are sexually dimorphic and are altered by severe stressors. One possibility is that LC-PAC1 receptors are highly expressed on BAT projecting neurons in male mice that undergo severe stressors than females or other control groups. Published work show that acute stressors increase *BAT Ucp1* activity (Nozu et al., 1992; Lkhagvasuren et al., 2011; Kataoka et al., 2014) and our studies corroborate those findings. We further show nuanced sex-specific differences in LC-PAC1 regulation of BAT activity and thermogenic program. However mechanistic aspects of BAT activity regulation via LC PAC1 remain to be elucidated. First, multiple physiological signals including LC-sympathetic pathways innervate BAT through multiple synapses (Cano et al., 2003; Almeida et al., 2004; Wiedmann et al., 2017). To gain a better understanding of anatomical link between LC-PAC1 and BAT, it will be important to determine how the PAC1 receptor expressing LC neurons project to BAT and whether LC-PAC1 alters BAT sympathetic tone to alter thermogenic program. Second, while LC expresses PAC1 receptors, the source/s of PACAPergic input to LC are unclear. The nucleus of the solitary tract (NTS), a structure known to express PACAP^99^ is a known major source of preganglionic input to the LC (Van Bockstaele et al., 1999). NTS integrates peripheral autonomic and endocrine signals associated with stressors, and regulates LC functions (Ulrich-Lai and Herman, 2009). The rostral ventrolateral medulla (RVLM) also contains high PACAPergic cells and innervate the LC, but their functions are related to cardiorespiratory functions which could influence metabolic functions indirectly. Thus, it will be important to discern if there are sex differences in NTS to LC projections.

In our current studies we did not measure the contribution of sex hormones in our sex-specific metabolic phenotype upon LC PAC1 ablation. While sex steroids, specifically estradiol, influence BAT activity (Heine et al., 2000; Pedersen et al., 2001; Karastergiou et al., 2012; Hoene et al., 2014; Valencak et al., 2017), future studies with ovariectomy/hormone replacement studies to determine roles of ovarian hormones (e.g., estradiol vs. progesterone) are needed. Besides estrous cycle, other studies will also be needed to determine if fat mass, lean mass, adult testicular hormones, gonadal hormonal surges in development, or the different complement of genes on the sex chromosomes (McCarthy et al., 2012) influence metabolic functions such as EE and RER and BAT activity between males and females.

Overall, our studies capture a granular detail of the LC in integrating severe stressor and metabolic signals via a genetically defined anatomy of LC via the PAC1 receptors. Our findings that LC-PAC1 neurons regulate metabolic responses under trauma-like stressors are important for further understanding the unique biology of LC via other systematic approach and consideration of sex differences. These studies are also important for growing studies in mapping brain and body interactions under severe stressors.

## Materials and methods

### Animals

All experimental procedures were conducted in accordance with guidelines set by the National Institutes of Health and the Institutional Animal Care and Use Committee at the Icahn School of Medicine at Mount Sinai. Mice were provided *ad libitum* access to food and water in a light- and temperature-controlled vivarium. Mice (3–4 months) were housed with no more than five mice per cage as littermates in a vivarium in a 12 h light:12 h dark cycle. Experiments were performed between 9 AM and 3 PM. The Adcyap1r1^loxP/loxP^ mouse line was utilized for all experiments. These mice were generated in a C57BL/6 background with a conditional knockout (KO) allele (PAC1^loxP/loxP^ mice) through the NIH-funded knockout mouse project (KOMP).

### Viruses

Mice were either injected with AAV2-hsyn-GFP-Cre for Cre-mediated deletion of PAC1 or AAV2-hsyn-GFP for control animals (UNC vector core).

### Cre mediated deletion of PAC1 receptors

To ablate PAC1 receptors in neurons, mice were secured in a stereotaxic apparatus under 2% isoflurane anesthesia and injected with rAAV2-hsyn-GFP-Cre to achieve neuronal deletion of PAC1 receptors. Control mice were injected with rAAV2-hsyn-eGFP. Mice were injected with 0.3 μl of virus bilaterally into the LC using coordinates (LM: ±1.0; AP: −5.4; DV: 4.2 from Bregma). Virus was microinfused into the LC with a 10 μl Hamilton Syringe fitted with a 1 mm glass pipette with no filament at a rate of 0.2 μl/min. After completion of infusion the glass pipette was left in position for 10 min to allow for diffusion. Using the glass pipettes that are commonly used for electrophysiological recordings with single cell resolution allowed confining the virus infusion to the LC. Using this method, we have previously targeted structures that are smaller than LC (Rajbhandari et al., 2021). Immediately after surgery, mice were given *ad libitum* access to a 0.5/0.1 mg/kg Sulfamethoxazole/Trimethoprim solution in drinking water for 5 days. The antibiotic regimen is standard procedure to prevent infections after surgeries. Since this is administered for 5 days after surgeries and the experimental procedure do not start until 3 weeks later, we do not expect it to have effects on metabolic functions. Mice also received a subcutaneous injection of the anti-inflammatory drug Rimadyl (5 mg/kg) immediately after, and 1 day following surgery. Mice were allowed 21 days after surgery prior to behavioral testing, which allowed for viral expression sufficient for Cre-mediated deletion of PAC1 receptors in LC neurons.

### Measurement of fear-related behavior

#### Conditioning apparatus

Mice were run individually in sound and light attenuated conditioning boxes (Med Associates Inc., Georgia, VT, USA) (Figure 1). The boxes were equipped with Near Infra-Red Video Fear Conditioning System and could be configured to represent different contexts by changing the internal structure, illumination, and odor. Context A (28 cm × 21 cm × 21 cm) had a clear Plexiglas back wall, ceiling, and front door with aluminum sidewalls visibly illuminated with a white light. It also had a grid floor with evenly spaced stainless-steel rods. Beneath the grid floor, in Context A, was an aluminum tray with a paper towel scented with 50% Windex. The floor in context A was connected to a scrambled foot shock generator. Context B (28 cm × 21 cm × 21 cm) had a clear plexiglass door with red walls illuminated with red colored LED light emitting from the top of the chamber. Context B also contained a grid floor connected to a scrambled foot shock generator, but beneath the grid floor was a paper towel scented with a 1% acetic acid solution.

#### Measure of freezing

Freezing is a complete lack of movement except for respiration (Fanselow, 1980). Freezing was measured using Ethovision software that performed real-time video recordings at 18 frames per second. With this program, adjacent frames are compared to provide the grayscale change for each pixel and the sum of pixels changing from one frame to the next constitutes a momentary activity score. To account for video noise and to approximate scoring by a trained human observer a threshold is set at 0.02 activity units so that an instance of freezing is counted when that the activity score remains below this threshold for 1 s (Anagnostaras et al., 2001). Percentage freezing = Freezing Time/Total Time × 100 for a period of interest. Data are presented as mean percentages (±SEM).

#### Behavioral design

We designed our behavioral tests to capture memory of the stressor. This design was chosen mainly because LC has been shown to be important for fear generalization (Soya et al., 2017).

##### Acute stress paradigm

After mice recovered from surgery an acute stress paradigm was used. On day 1, mice were transported to the behavioral testing area in their clear plastic home cage and placed in the chambers set up in Context A. Mice were then exposed to 10 random foot shocks (1 mA) over the period of 60 min. This stressor has been shown to produce long-lasting effects (Rau and Fanselow, 2009). Mice were transported to the laboratory together in their home cages. For the behavioral experiments male mice were always ran before females and chambers were thoroughly cleaned between mice to avoid effects of pheromones on behavior.

#### Memory of stressor test

On day 2 mice were transported to the behavioral testing room in a round, opaque plastic container which was distinct from their home cage and placed in chambers set up in Context B. The animals were placed in a different Context (B) for four minutes and thirty seconds. Mice were allowed to explore Context B for 4 min and time spent freezing was measured for this time-period.

### Indirect calorimetry using metabolic chambers

Three days after administration of the acute stress paradigm, mice were placed in in indirect calorimetry chambers (Sable Promethion) for 72 h. Data collected from indirect calorimetry included oxygen consumption, carbon dioxide production, energy expenditure (EE), respiratory exchange ratio (RER), energy balance, food and water intake, locomotor activity, and body mass. This information highlights changes in metabolic physiology after severe stressor as a function of presence/absence of LC-PAC1. The first 24 h of indirect calorimetry data was excluded from analysis to allow mice to habituate to the novel environment. Body weight was used as statistical covariates for the analysis of some indirect calorimetry measurements in the metabolic chambers such as oxygen consumption, carbon dioxide production, EE, and food and water intake. RER value and locomotion were analyzed without a covariate as they are known to be measures independent of body weight. The RER value, which is a ratio of the volume carbon dioxide produced over the volume of oxygen consumed, show if the predominant source of energy is fat or carbohydrate after stressor. A higher RER value denotes carbohydrate as the primary source of energy being utilized, while a lower RER value indicates fat as a fuel source. We analyzed the data with CalR (Mina et al., 2018) that considers activity, food intake and other parameters allowing us to derive accurate indirect calorimetry values.

### Tissue harvests

Three days after indirect calorimetry mice were food deprived for 4 hours and anesthetized with isoflurane and rapidly decapitated. Brain and BAT were collected and rapidly frozen and stored in a −80 ^°^C freezer. Brain tissue with LC were used to confirm injection sites and loss of PAC1 using RNAScope *in situ* hybridization routinely used in our lab^58^.

### RNAscope *in situ* hybridization

The brains were sectioned at 20 μm in a cryostat at −20^°^C and slices containing the LC were collected on Fisherbrand Superfrost Plus microscope slides (Thermo Fisher Scientific) and stored at −80 ^°^C. Deletion of PAC1 receptors was verified using RNAscope for analyzing expression of RNA tissue sections (ACD Biotechne). Briefly, we performed *in situ* hybridization steps following RNAscope^®^ 2.5 HD HD Assay - RED protocol for fresh frozen sections. After completion of the labeling, sections were cover-slipped using Prolong Gold (Thermo Fisher Scientific) with 4’,6-diamidino-2- phenylindole (DAPI) and the edges were sealed with clear nail polish. PAC1 mRNA quantification was carried out in sections containing the LC that were captured with a 20X objective on a Zeiss AxioImager Z2M with ApoTome. Analysis of PAC1 mRNA was conducted using FIJI software. For quantification, mean fluorescence of PAC1 mRNA labeled with mCherry inside a standard section inside the LC was assessed.

### RNA purification, cDNA synthesis, and RT-qPCR

RNA was isolated from BAT using phenol-chloroform extraction. After isolated, the RNA pellet was washed and resuspended in diethyl pyrocarbonate (DEPC) water at a concentration of 200 ng/μL. RNA samples were reversely transcribed to cDNA using a high-capacity cDNA reverse transcription kit (Applied Biosystems). Real Time qPCR was performed by using a real time PCR SYBR green master mix (Diagenode) and primers for *Ucp1*, peroxisome proliferator-activated receptor (*PPARa*), cytochrome c oxidase, subunit VIIIb (*Cox8b*), elongation of very long chain fatty acids-like 3 (*Elovl3*), Calsyntenin 3 (*Clstn3*), peroxisome proliferator-activated receptor-gamma coactivator-1 alpha (*PPARGC1a*). Samples were run and analyzed on a Quantstudio 5 (Applied Biosystems). The qPCR targets were normalized to the expression of the housekeeping gene *36B4*.

### Microscopy for all experiments

The tissue sections were analyzed using a Zeiss AxioImager Z2M with ApoTome microscope. Images were analyzed with Fiji image processing software (NIH, Bethesda, MD, USA; RRID:SCR_002285). Mean fluorescent intensity of PAC1 RNA was measured on a section of LC tagged with GFP.

### Experimental design and the statistical analyses

We measured freezing for the first 4 min of the session on day 2 of the acute stress paradigm. For the behavioral and qPCR experiments, a two-way analysis of variance (ANOVA) was used to measure differences in means with two between sex and condition (virus and stress/control) factors. Significant effects indicated by the ANOVA were further analyzed with a post-hoc Holm-Sida’s post-hoc analysis. For metabolic experiments, a two-way analysis of variance (ANOVA) was performed on measurements not associated with mass (i.e., respiratory exchange ratio (RER) and locomotor activity) and analysis of covariance (ANCOVA) with total mass as a covariate for measurements that are associated with mass [i.e., oxygen consumption, carbon dioxide production, food and water consumption, and energy expenditure (EE)]. The level of significance used for all analyses was *p* < 0.05.

## Data availability statement

The original contributions presented in this study are included in the article/Supplementary material, further inquiries can be directed to the corresponding author.

## Ethics statement

This animal study was reviewed and approved by IACUC.

## Author contributions

SD, NO, SS, SP, and FM ran the experiments. SD, PR, and AR wrote the manuscript. All authors contributed to the article and approved the submitted version.

## Supplementary material

Supplementary Figure 1 **(A–H)** Oxygen consumption (VO2, ml/h) **(A,B)**, carbon dioxide production (VCO2, ml/h) **(C,D)**, respiratory exchange ratio (RER) **(E,F)**, energy expenditure (EE) **(G,H)**, food consumption (kcal/h) **(I,J)**, and locomoter activity (beam breaks/h) **(K,L)**, **(C)** of male and female mice analyzed in Sable Promethion metabolic chambers (12 h light/dark cycle, first 24 h total duration, white bar represent light cycle and grey bar represent night cycle). For each of these variables a line graph comparing all four groups (hSyn-Cre S, hSyn-Cre NS, hSyn-GFP S, and hSyn-GFP NS) are displayed. *N* = 9–10/group. GFP NS (hSyn-GFP No Shock), GFP S (hSyn-GFP Shock), CRE NS (hSyn-Cre No Shock), and CRE S (hSyn-Cre Shock). No significant differences were observed in these variables.

Supplementary Figure 2 **(A–D)** No significant differences were observed in oxygen consumption (VO2, ml/h) **(A)**, carbon dioxide production (VCO2, ml/h) **(B)**, locomotor activity (beam breaks/h), locomotor activity (beam breaks/h) **(C)**, and food consumption (kcal/h) **(D)** of male mice. **(E–G)** Similarly, no significant differences in oxygen consumption (VO2, ml/h) **(E)**, carbon dioxide production (VCO2, ml/h) **(F)**, and locomotor activity (beam breaks/h) **(G)** were observed in females. However, stressed females with LC-PAC1 deletion showed a significant increase in food consumption (kcal/h) compared to stressed females with intact LC-PAC1 **(H)**. All data were collected using Sable Promethion metabolic chambers (12 h light/dark cycle, 48 h total duration, white bar represent light cycle and gray bar represent night cycle). For each of these variables a line graph and bar graph comparing all four groups (hSyn-Cre S, hSyn-Cre NS, hSyn-GFP S, and hSyn-GFP NS) as well as a line graph comparing stressed groups (hSyn-Cre S and hSyn-GFP S) are displayed. *N* = 9–10/group. **p* < 0.05; ^**^*p* < 0.01. GFP NS, hSyn-GFP No Shock; GFP S, hSyn-GFP Shock; CRE NS, hSyn-Cre No Shock; CRE S, hSyn-Cre Shock.

Supplementary Figure 3 **(A–H)** Real-time qPCR of indicated genes from male and female BATs of indicated mouse groups. No significant differences were observed in these variables. *N* = 9-10/group. *PPARa*, peroxisome proliferator-activated receptor; *Elovl3*, elongation of very long chain fatty acids-like 3; *Dio*, idothyronine deiodinase 2; *Clstn3*, Calsyntenin 3.

Supplementary Table 1 Generalized linear model (GLM) ANCOVA and ANOVA tests for the listed parameters from male mice housed in metabolic chambers.

Supplementary Table 2 Generalized linear model (GLM) ANCOVA and ANOVA tests for the listed parameters from female mice housed in metabolic chambers.
