# Supplementary figures and images for: Sexually dimorphic role of the locus coeruleus PAC1 receptors in regulating acute stress-associated energy metabolism
Source: Front Behav Neurosci. 2022 Oct 5;16:995573. doi: 10.3389/fnbeh.2022.995573 (PMC9580361; doi:10.3389/fnbeh.2022.995573)

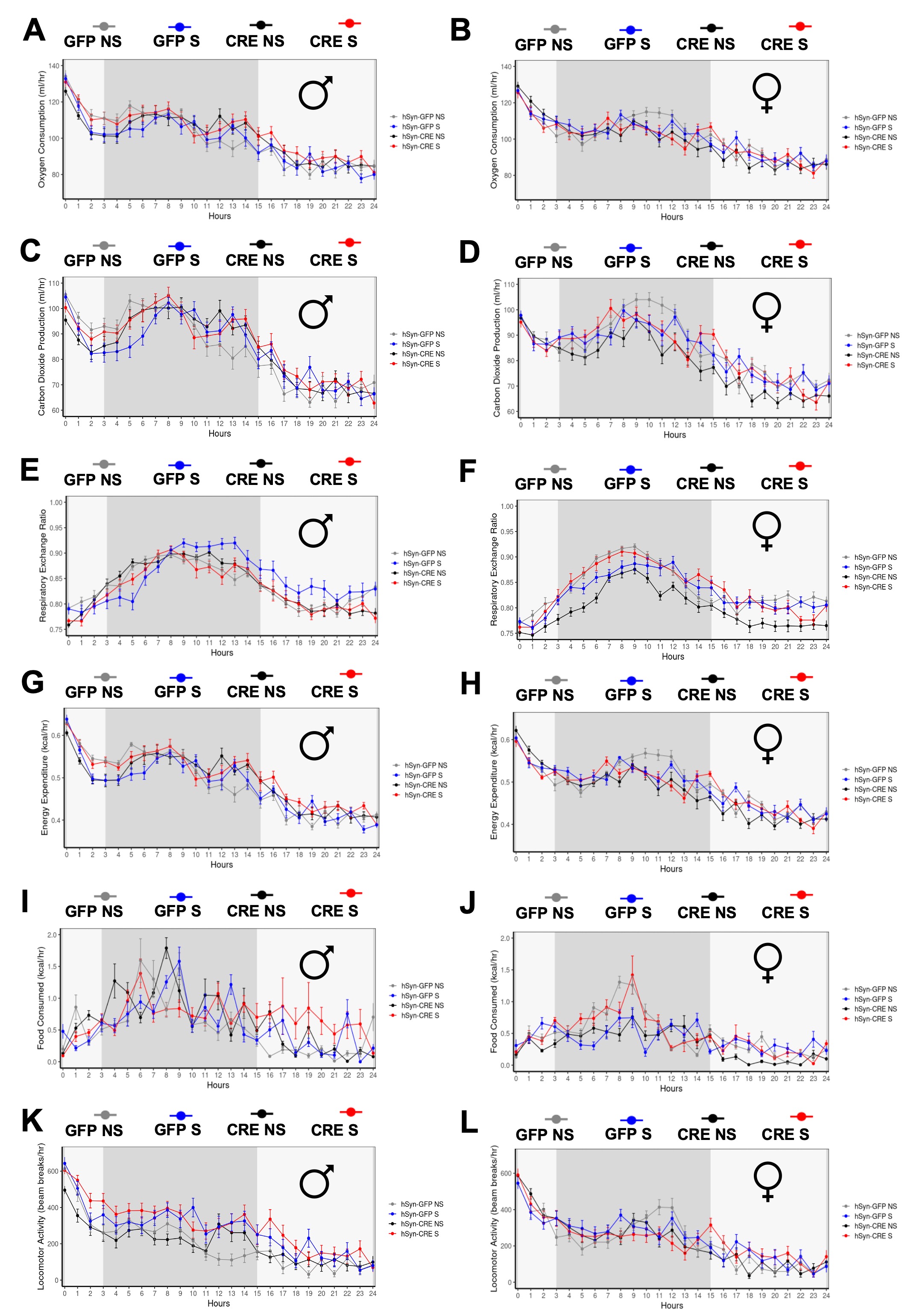

Supplement: Supplementary Figure 1 — (A–H) Oxygen consumption (VO2, ml/h) (A,B), carbon dioxide production (VCO2, ml/h) (C,D), respiratory exchange ratio (RER) (E,F), energy expenditure (EE) (G,H), food consumption (kcal/h) (I,J), and locomoter activity (beam breaks/h) (K,L), (C) of male and female mice analyzed in Sable Promethion metabolic chambers (12 h light/dark cycle, first 24 h total duration, white bar represent light cycle and grey bar represent night cycle). For each of these variables a line graph comparing all four groups (hSyn-Cre S, hSyn-Cre NS, hSyn-GFP S, and hSyn-GFP NS) are displayed. N = 9–10/group. GFP NS (hSyn-GFP No Shock), GFP S (hSyn-GFP Shock), CRE NS (hSyn-Cre No Shock), and CRE S (hSyn-Cre Shock). No significant differences were observed in these variables. [file Image_1.jpg]

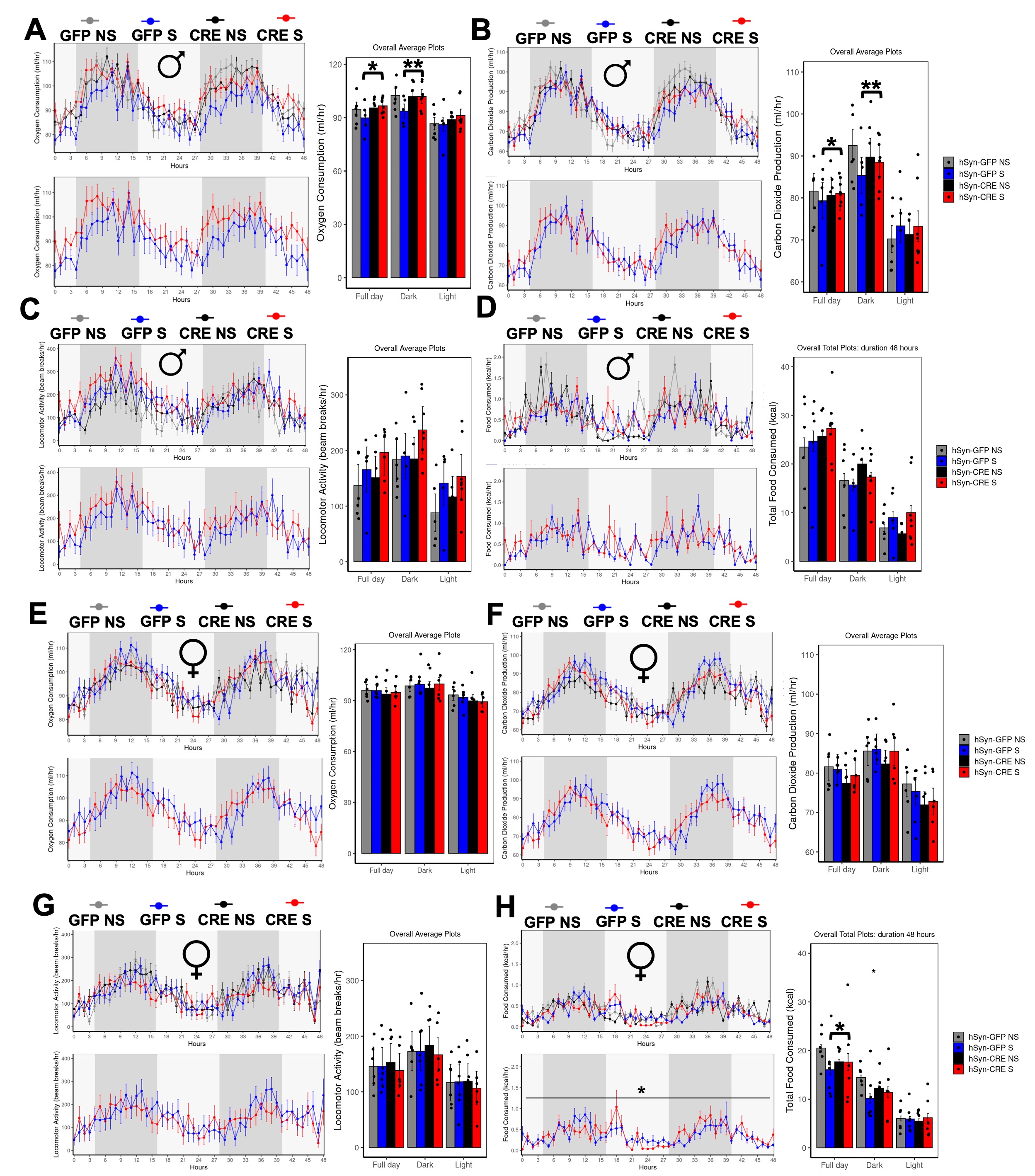

Supplement: Supplementary Figure 2 — (A–D) No significant differences were observed in oxygen consumption (VO2, ml/h) (A), carbon dioxide production (VCO2, ml/h) (B), locomotor activity (beam breaks/h), locomotor activity (beam breaks/h) (C), and food consumption (kcal/h) (D) of male mice. (E–G) Similarly, no significant differences in oxygen consumption (VO2, ml/h) (E), carbon dioxide production (VCO2, ml/h) (F), and locomotor activity (beam breaks/h) (G) were observed in females. However, stressed females with LC-PAC1 deletion showed a significant increase in food consumption (kcal/h) compared to stressed females with intact LC-PAC1 (H). All data were collected using Sable Promethion metabolic chambers (12 h light/dark cycle, 48 h total duration, white bar represent light cycle and gray bar represent night cycle). For each of these variables a line graph and bar graph comparing all four groups (hSyn-Cre S, hSyn-Cre NS, hSyn-GFP S, and hSyn-GFP NS) as well as a line graph comparing stressed groups (hSyn-Cre S and hSyn-GFP S) are displayed. N = 9–10/group. *p < 0.05; **p < 0.01. GFP NS, hSyn-GFP No Shock; GFP S, hSyn-GFP Shock; CRE NS, hSyn-Cre No Shock; CRE S, hSyn-Cre Shock. [file Image_2.jpg]

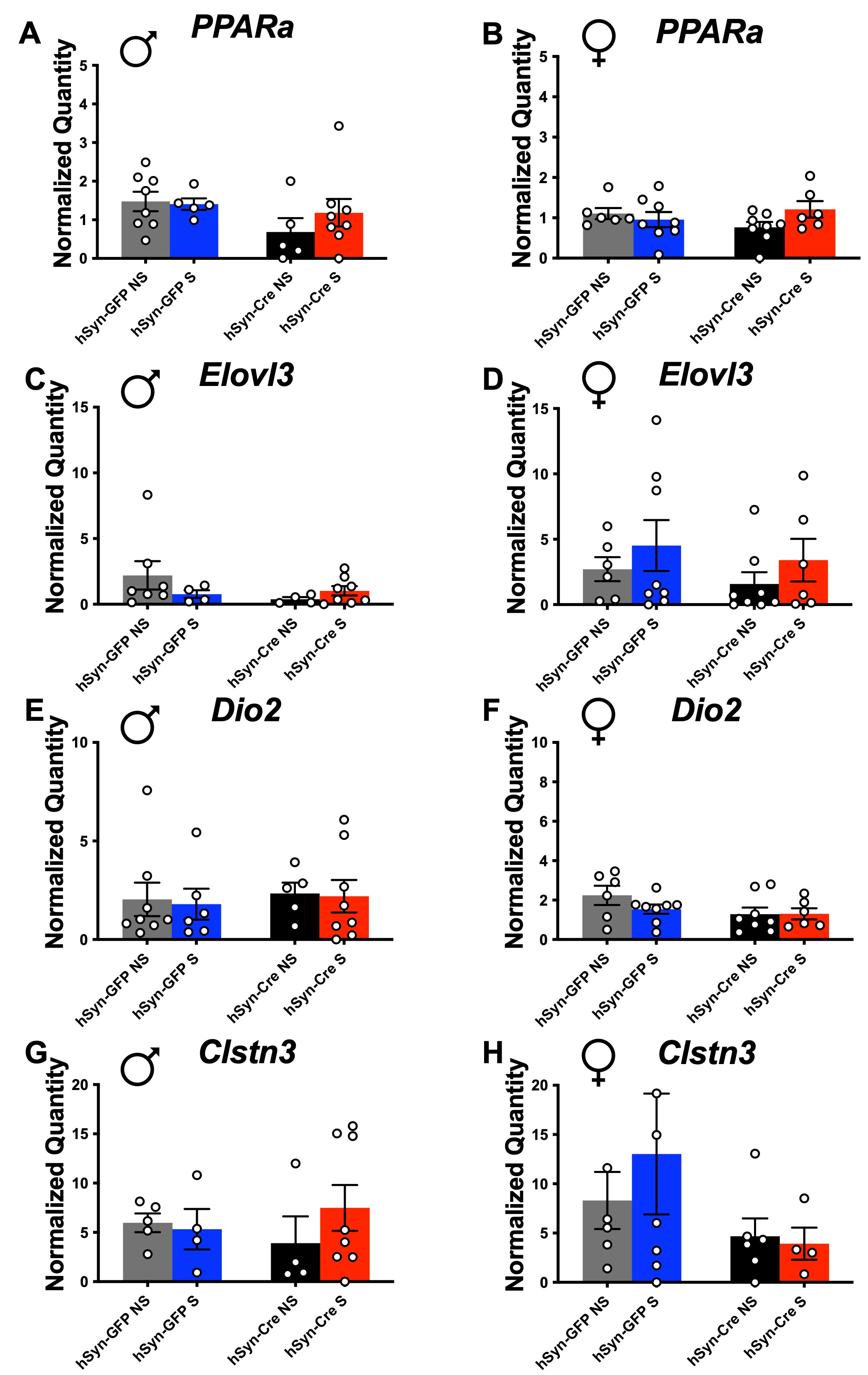

Supplement: Supplementary Figure 3 — (A–H) Real-time qPCR of indicated genes from male and female BATs of indicated mouse groups. No significant differences were observed in these variables. N = 9-10/group. PPARa, peroxisome proliferator-activated receptor; Elovl3, elongation of very long chain fatty acids-like 3; Dio, idothyronine deiodinase 2; Clstn3, Calsyntenin 3. [file Image_3.jpg]
