# Supplementary material for: Sexually dimorphic role of the locus coeruleus PAC1 receptors in regulating acute stress-associated energy metabolism
Source: Front Behav Neurosci. 2022 Oct 5;16:995573. doi: 10.3389/fnbeh.2022.995573 (PMC9580361; doi:10.3389/fnbeh.2022.995573)
Supplement: Supplementary Table 1 — Generalized linear model (GLM) ANCOVA and ANOVA tests for the listed parameters from male mice housed in metabolic chambers. [file Table_1.pdf]

Males									
**GLM (hSyn-GFP S vs hSyn-CRE S)**									
	Full Day			Light			Dark		
**Effect**	Mass	Group	Interaction	Mass	Group	Interaction	Mass	Group	Interaction
**Food Consumed (kcal/hr)**	0.1325	0.5706		0.6391	0.5396		0.0044**	0.9295	
**Water Consumed (ml/hr)**	0.7864	0.3573		0.2949	0.4523		0.6199	0.4696	
**Energy Expenditure (kcal/hr)**	0.5566	0.0307*	0.0366*	0.0100*	0.0014**	0.0016**	<0.001***	0.2787	
**Oxygen Consumption (ml/hr)**	0.6318	0.0379*	0.0467	0.0110*	0.0012**	0.0014**	<0.001***	0.1703	
**Carbon Dioxide Production (ml/hr)**	0.3912	0.0371*	0.0375*	0.0119*	0.0043**	0.0043**	<0.001***	0.7695	
**GLM (hSyn-CRE NS vs hSyn-CRE S)**									
	Full Day			Light			Dark		
**Effect**	Mass	Group	Interaction	Mass	Group	Interaction	Mass	Group	Interaction
**Food Consumed (kcal/hr)**	0.1325	0.5569		0.6391	0.2614		0.0044**	0.5881	
**Water Consumed (ml/hr)**	0.7864	0.7367		0.2949	0.7689		0.6199	0.4240	
**Energy Expenditure (kcal/hr)**	0.5566	0.0395*	0.0436*	0.0100*	0.0047**	0.0049**	<0.001***	0.3327	
**Oxygen Consumption (ml/hr)**	0.6318	0.0568	0.0627	0.0110*	0.0044	0.0046**	<0.001***	0.3193	
**Carbon Dioxide Production (ml/hr)**	0.3912	0.0254*	0.0272*	0.0119*	0.0093**	0.0095**	<0.001***	0.5066	
**GLM (hSyn-GFP NS vs hSyn-CRE S)**									
	Full Day			Light			Dark		
**Effect**	Mass	Group	Interaction	Mass	Group	Interaction	Mass	Group	Interaction
**Food Consumed (kcal/hr)**	0.1325	0.5947		0.6391	0.6148		0.0044**	0.8498	
**Water Consumed (ml/hr)**	0.7864	0.5936		0.2949	0.6454		0.6199	0.7578	
**Energy Expenditure (kcal/hr)**	0.5566	0.0051**	0.0063**	0.0100*	0.0032**	0.0033**	<0.001***	0.0215*	
**Oxygen Consumption (ml/hr)**	0.6318	0.0070**	0.0087**	0.0110*	0.0025**	0.0026**	<0.001***	0.0193*	
**Carbon Dioxide Production (ml/hr)**	0.3912	0.0065**	0.0073**	0.0119*	0.0113*	0.0109*	<0.001***	0.0903	

Males									
**ANOVA**									
	Full Day			Light			Dark		
**Effect**	(hSyn-GFP S vs hSyn-CRE S)	(hSyn-CRE NS vs hSyn-CRE S)	(hSyn-GFP NS vs hSyn-CRE S)	(hSyn-GFP S vs hSyn-CRE S)	(hSyn-CRE NS vs hSyn-CRE S)	(hSyn-GFP NS vs hSyn-CRE S)	(hSyn-GFP S vs hSyn-CRE S)	(hSyn-CRE NS vs hSyn-CRE S)	(hSyn-GFP NS vs hSyn-CRE S)
**Pedestrian Locomotion (m)**	0.0764	0.0641	0.0055**	0.1415	0.0931	0.0075**	0.0328	0.0437*	0.0046**
**Total Distance in Cage (m)**	0.0807	0.0532	0.0043**	0.1520	0.0819	0.0061**	0.0335*	0.0335*	0.0035**
**Respiratory Exchange Ratio**	0.0282*	0.7783	0.2041	0.0503	0.9779	0.4972	0.0764	0.6197	0.1392
**Locomotor Activity (beam breaks/hr)**	0.3360	0.1784	0.0394*	0.6706	0.3614	0.0652	0.2383	0.1519	0.0704
