# Supplementary material for: Sexually dimorphic role of the locus coeruleus PAC1 receptors in regulating acute stress-associated energy metabolism
Source: Front Behav Neurosci. 2022 Oct 5;16:995573. doi: 10.3389/fnbeh.2022.995573 (PMC9580361; doi:10.3389/fnbeh.2022.995573)
Supplement: Supplementary Table 2 — Generalized linear model (GLM) ANCOVA and ANOVA tests for the listed parameters from female mice housed in metabolic chambers. [file Table_2.pdf]

Females

GLM (hSyn-GFP S vs hSyn-CRE S)

	Full Day			Light			Dark		
**Effect**	Mass	Group	Interaction	Mass	Group	Interaction	Mass	Group	Interaction
**Food Consumed (kcal/hr)**	0.0036**	0.0118*	0.0117*	0.0200*	0.0561	0.0501	0.0314*	0.0556	0.0589
**Water Consumed (ml/hr)**	0.4387	0.2744		0.8835	0.5532		0.2986	0.1970	
**Energy Expenditure (kcal/hr)**	0.0048**	0.5865		0.0091**	0.3172		0.0578	0.9581	
**Oxygen Consumption (ml/hr)**	0.0072**	0.6148		0.0085**	0.3114		0.0620	0.9636	
**Carbon Dioxide Production (ml/hr)**	0.2968	0.6557	0.7126	0.0255*	0.4038		0.0693	0.9356	

GLM (hSyn-CRE NS vs hSyn-CRE S)

	Full Day			Light			Dark		
**Effect**	Mass	Group	Interaction	Mass	Group	Interaction	Mass	Group	Interaction
**Food Consumed (kcal/hr)**	0.0036**	0.1375	0.1506	0.0200*	0.0298*	0.0328*	0.0314*	0.7727	0.7962
**Water Consumed (ml/hr)**	0.4387	0.6368		0.8835	0.5614		0.2986	0.7920	
**Energy Expenditure (kcal/hr)**	0.0048**	0.9644		0.0091**	0.6111		0.0578	0.7185	
**Oxygen Consumption (ml/hr)**	0.0072**	0.8920		0.0085**	0.5483		0.0620	0.7887	
**Carbon Dioxide Production (ml/hr)**	0.2968	0.0410*	0.0423*	0.0255*	0.8885		0.0693	0.4300	

GLM (hSyn-GFP NS vs hSyn-CRE S)

	Full Day			Light			Dark		
**Effect**	Mass	Group	Interaction	Mass	Group	Interaction	Mass	Group	Interaction
**Food Consumed (kcal/hr)**	0.0036**	0.0048**	0.0035**	0.0200*	0.0199*	0.0197*	0.0314*	0.0367*	0.0258*
**Water Consumed (ml/hr)**	0.4387	0.2988		0.8835	0.5962		0.2986	0.2339	
**Energy Expenditure (kcal/hr)**	0.0048**	0.7581		0.0091**	0.4196		0.0578	0.8192	
**Oxygen Consumption (ml/hr)**	0.0072**	0.8281		0.0085**	0.4445		0.0620	0.7906	
**Carbon Dioxide Production (ml/hr)**	0.2969	0.4193	0.3714	0.0255*	0.3982		0.0693	0.9969	

Females

ANOVA

	Full Day			Light			Dark		
**Effect**	(hSyn-GFP S vs hSyn-CRE S)	(hSyn-CRE NS vs hSyn-CRE S)	(hSyn-GFP NS vs hSyn-CRE S)	(hSyn-GFP S vs hSyn-CRE S)	(hSyn-CRE NS vs hSyn-CRE S)	(hSyn-GFP NS vs hSyn-CRE S)	(hSyn-GFP S vs hSyn-CRE S)	(hSyn-CRE NS vs hSyn-CRE S)	(hSyn-GFP NS vs hSyn-CRE S)
**Pedestrian Locomotion (m)**	0.4479	0.1318	0.7027	0.4501	0.1291	0.7971	0.4888	0.1673	0.6401
**Total Distance in Cage (m)**	0.5556	0.2160	0.7991	0.5322	0.2371	0.9261	0.6080	0.2129	0.6984
**Respiratory Exchange Ratio**	0.9555	0.5859	0.5793	0.8351	0.5993	0.5547	0.9100	0.6589	0.7126
**Locomotor Activity (beam breaks/hr)**	0.6531	0.5541	0.7130	0.5917	0.7995	0.8429	0.7498	0.4812	0.6713
